# A Biometric Identification for Multi-Modal Biomedical Signals in Geriatric Care

**DOI:** 10.3390/s24206558

**Published:** 2024-10-11

**Authors:** Yue Che, Lingyan Du, Guozhi Tang, Shihai Ling

**Affiliations:** 1 School of Automation and Information Engineering, Sichuan University of Science and Engineering, Zigong 643000, China; 2 Artificial Intelligence Key Laboratory of Sichuan Province, Sichuan University of Science and Engineering, Zigong 643000, China

**Keywords:** biosignals, multi-modal, fusion, human identification, machine learning

## Abstract

With the acceleration of global population aging, the elderly have an increasing demand for home care and nursing institutions, and the significance of health prevention and management of the elderly has become increasingly prominent. In this context, we propose a biometric recognition method for multi-modal biomedical signals. This article focuses on three key signals that can be picked up by wearable devices: ECG, PPG, and breath (RESP). The RESP signal is introduced into the existing two-mode signal identification for multi-mode identification. Firstly, the features of the signal in the time–frequency domain are extracted. To represent deep features in a low-dimensional feature space and expedite authentication tasks, PCA and LDA are employed for dimensionality reduction. MCCA is used for feature fusion, and SVM is used for identification. The accuracy and performance of the system were evaluated using both public data sets and self-collected data sets, with an accuracy of more than 99.5%. The experimental data fully show that this method significantly improves the accuracy of identity recognition. In the future, combined with the signal monitoring function of wearable devices, it can quickly identify individual elderly people with abnormal conditions, provide safer and more efficient medical services for the elderly, and relieve the pressure on medical resources.

## 1. Introduction

Population aging is a common challenge facing the world [1]. The global population aged 65 and over will reach 761 million in 2021 and increase to 1.6 billion in 2025 [2]. The increasing longevity is closely linked to the healthcare and welfare of the elderly. At the same time, the array of adverse effects associated with aging are primarily intertwined with a country’s economic development. Healthy aging serves as a pivotal factor in addressing global responses to population aging [3]. The accelerating process of population aging has brought about many impacts on society. First, the soaring demand for hospital beds, medical equipment, and nursing staff has strained medical resources. Second, aging brings about changes in family structure. The function of family elderly care is becoming weaker and weaker, resulting in a sharp rise in the demand for auxiliary medical services such as nursing homes, home care, and intelligent elderly care equipment. In the aging society, rationally allocating medical resources and constructing a sound old-age service system has become a significant challenge. The elderly population often has multiple chronic diseases (diabetes, hypertension, heart disease, etc.), and there is a strong need for continuous health monitoring and condition management in nursing homes and home care settings. In this case, in-home care and elderly care mainly adopt the “time segment” model; the nursing staff provides nursing services in time segments rather than all-weather escorts. In nursing homes, one healthcare worker may be responsible for several elderly people at the same time, and elderly people who need special care (such as disabled and mentally retarded elderly) need more care resources. Twenty-four-hour one-on-one care is more difficult to achieve when medical resources are tight. The development and popularity of wearable devices such as smart wristbands, smartwatches, and health monitoring devices have increased public health awareness. All-weather access to physiological information and measurement of human vital signs, such as electrocardiogram (ECG), photoelectric plethysmography (PPG), and other signals, to achieve heart rate monitoring, sleep analysis, blood oxygen saturation measurement, etc., has become a necessary health detection function of wearable devices. Some wearable devices have reached medical-grade standards and can be used for telemedicine and patient monitoring. This helps older adults better manage their health and detect potential health problems early. In the future, in elderly care institutions or elderly home care scenarios, combined with the identification technology that wearable devices can collect signals (such as ECG, PPG, RESP), wearable devices can not only collect and monitor physiological data in real-time but also issue abnormal early warnings when the elderly are found to be abnormal and carry out elderly identification authentication. This will put data immediately into the hands of medical staff and family members in cases where elderly people live alone and nursing home resources are stretched, leading to inadequate care. This not only provides timely risk warning for the elderly but also reduces the delay in diagnosis. It also reduces the workload of checking personnel information for medical staff and reduces the work pressure and burden of medical staff. In general, combining biomedical signal signature identification with wearable devices can not only reduce the reliance on manpower but also ensure that the elderly receive continuous care and security. In promoting the development of personalized medicine and efficient nursing, the high security, accuracy, and convenience of biomedical signal feature identification has a wide range of practical applications in geriatric care and medical health.

The biomedical signal is an inherent and long-term unchanged reflection of the human body’s physiological state, with its vital advantage lying in the perception of life [4]. It possesses significant specificity, non-reproducibility, and uniqueness, which provides substantial benefits over traditional biometric recognition methods. However, single biomedical signal recognition technology may encounter issues such as limited expressiveness, severe signal interference, and low recognition rates that can potentially impact the accuracy of identity recognition to some extent. Multi-modal biomedical signal recognition addresses these problems encountered by single-mode approaches [5]. Researchers have employed various methods for classifying and identifying these signals while utilizing diverse algorithms to enhance system efficiency and performance. Everson et al. [6] proposed a new deep learning framework (BiometricNET) for PPG signal identification. The model uses four deep neural networks: two CNN layers linked to two LSTM layers, followed by a dense output layer. The proposed network configuration was evaluated using the TROIKA database collected from 12 participants participating in physical activity, achieving 96% cross-validation accuracy. Li et al. [7] proposed a cascaded convolutional neural network and used this network for identification based on ECG biomedical signals. Two convolutional neural networks are designed; the first one is called F-CNN for ECG feature extraction, and the second one is called M-CNN for biometric comparison and recognition. Performance was evaluated across five public data sets of PhysioNet, with an average recognition rate of 94.3%. The reliability and stability of biomedical signals in identity recognition are discussed in the single biomedical signal identification research, which provides the theoretical basis and practical support for the subsequent research and development of biomedical signals in multi-modal identity recognition technology.

Hammad et al. [8] utilized a convolutional neural network (CNN) to integrate electrocardiogram (ECG) and fingerprint data at varying degrees, proposing two multi-modal biometric identification systems with feature-level fusion and decision-level fusion. They employed QG-MSVM classification for identity verification. The experimental results demonstrate that the proposed multi-modal system outperforms previous approaches regarding overall performance, efficiency, robustness, and reliability. Farhad Ahamed et al. [9] introduced time domain and joint time–frequency domain feature extraction methods to extract informative features from ECG and PPG and fused ECG-PPG signals. These methods were evaluated on five publicly available datasets from Physionet and Mendeley data repositories. The findings reveal that the multi-modal fusion authentication model achieves an accuracy of 99.8% with an error rate of 0.16%, surpassing single-mode approaches in accuracy and reliability. Through algorithmic calculations and mathematical equations, Bastos et al. [10] determined the authentication error rate of ECG and PPG signals collected non-invasively from 53 individuals using acquisition methods. They discussed the feasibility of utilizing ECG and PPG signals for biometric identification. Chayma Yaacoubi et al. [11] employed a combination of deep neural networks, including CNNs and recurrent neural networks (RNNs), and tested ECG and PPG data from 12 subjects in the ROIKA database. Their study concluded that fusing these two datasets while utilizing RNN’s gated recurrent unit (GRU) could enhance accuracy by up to 94%. El Rahiem et al. [12] used multiple typical correlation analysis (MCCA) to fusion ECG and finger veins after feature extraction by CNN. Five famous machine learning classifiers, namely, support vector machine (SVM), K-nearest neighbor (KNN), Random Forest (RF), Naive Bayes (NB), and artificial neural network (ANN), are used to identify and authenticate the extracted features. The authentication accuracy of the proposed multi-modal system using the KNN classifier and MCCA feature fusion is improved by 10% on average compared with other machine learning algorithms. Ebrahim et al. [13] combined fingerprint, ECG, and face image data using feature level and fractional level fusion, ensuring that the proposed model achieved similar performance (more than 80%) with incomplete data (missing one of the three) and that the proposed model achieved better generalization on the baseline data set used. GirishRaoSalankeN S [14] explored the possibility of incorporating electrocardiograms as part of multi-modal biometrics, fusing it with traditional biometrics (such as fingerprints), and proposed a multi-modal biometrics system with an accuracy rate of 98%, a false acceptance rate of 2%, and a false rejection rate of almost 0%.

**Figure 1:**
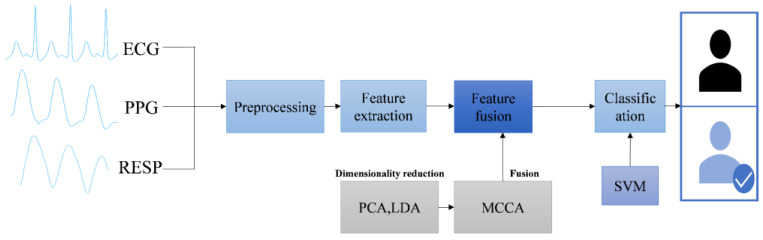
Block diagram of multi-modal identification.

The existing literature shows that some multi-modal biomedical signal feature identification is based on single signals such as ECG, PPG, and EEG [15,16]. Still, single-modal biometric identification is vulnerable to attacks such as theft and impersonation. Some are based on multi-modal signals, such as ECG and PPG bimodal feature identification and biomedical signals combined with other biometric identification (such as fingerprints, faces, and finger veins) [17,18]. Compared with the other literature, in view of the difficulty and integration advantages of wearable devices to collect physiological signals, the technology can be combined with wearable devices (such as smartwatches and smart bracelets) in the future on the basis of the existing ECG and PPG signal dual-mode recognition, the first time to introduce respiratory signal (RESP) as a third biomedical signal for multi-mode signal recognition. And in terms of data sets, three kinds of biomedical signals from wearable devices were collected through the laboratory’s Pclab-801 embedded biomedical electronic laboratory box. We performed experiments on both public and self-collected datasets, demonstrating the effectiveness of this approach for biometric authentication compared to most previous work on recognizing these biomedical signals. The whole process is divided into four essential stages: signal preprocessing, feature extraction, dimensionality reduction fusion, and identity recognition. Firstly, the biomedical signals are preprocessed to extract the features. Aiming at the dimensionality disaster caused by multi-modal biomedical signal fusion, PCA and LDA feature dimensionality reduction are performed to improve the accuracy of three signal feature matrices. The best fusion feature matrix is obtained by MCCA three-mode fusion. Finally, a support vector machine is used for identification, as shown in Figure 1.

To verify the feasibility and validity of this method, firstly, different data sets from other sources (including self-collected and published on the Internet) should be included in the study to ensure that the sources of the data sets are different. Second, research methods should show exemplary test results on various data sets, ensuring no bias in other data sets. Third, when designing a process, one of the most important stages is to choose the most appropriate indicator. Since all data sets are relatively balanced, we decided to select an identification accuracy to estimate the performance of the method, and the accuracy can be expressed as follows:

(1) $$Accuracy=\frac{TP+TN}{TP+TN+FP+FN}$$

where TP is the number of correct predictions for the positive sample,TN is the number of correct predictions for the adverse sample,FN is the number of false predictions for the positive sample, and FP is the number of all false predictions for the negative sample.

## 2. Materials and Methods

### 2.1. Database

The MIMIC database [19] used in this paper is a publicly accessible multi-parameter critical care database provided by the Computational Physiology Laboratory of Massachusetts Institute of Technology. Biomedical data such as ECG, Pleth, arterial blood pressure (ABP), RESP, and oxygen saturation (SpO2) were collected from the ICU for more than 69 patients. The data in each case included signals and periodic measurements from bedside monitors and clinical data from patient records, almost all of which were at least 20 h long, and many were 40 h or more. The database contains real-time signals and related data for nearly 200 patient days. In verifying and screening individual data in the MIMIC database, the following data screening criteria are applied: First, data integrity and consistency; each individual’s data in the MIMIC database do not contain a record of three medical signals—ECG, PPG, and RESP. Screening can ensure that each individual’s data includes the necessary medical signals to ensure the integrity and consistency of the data. Second, data quality; where there is continuous signal loss and interruption in the record, screening can eliminate individual data with excessive data loss or poor-quality data. After standard screening, ECG, PPG, and RESP signal data of 25 patients were finally selected to form the experimental data set. This data source comes from PhysioNet [20].

**Figure 2:**
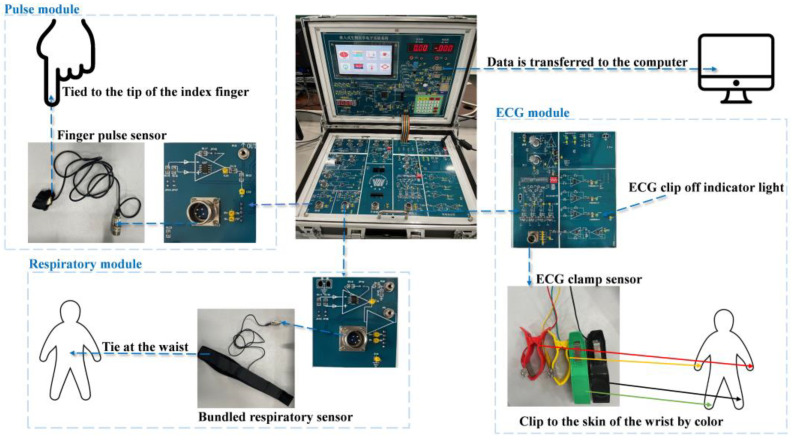
Biomedical signal acquisition experiment diagram.

In this paper, ECG, PPG, and RESP were collected using a Pclab-801 embedded biomedical electronic experiment box (Made in Beijing Microster Technology, Beijing, China). The Pclab-801 embedded biomedical electronic experiment box is used to collect the biomedical signals of the subjects through energy conversion, signal acquisition, amplification, filtering A/D conversion, and single-chip processing, and the signal waveform changes can be observed in real-time on the LCD. The biomedical signal acquisition experiment is shown in Figure 2.

When the surrounding environment is less disturbed and the subject is quiet and relaxed, the subject is asked to remove shoes, socks, and tight clothing to ensure that the collected electrodes are close to the skin. The subject is allowed to lie flat on the bed, the ECG clip-on sensor in accordance with the corresponding color on both arm, wrist, and ankle joints, forming limb leads. When the ECG clip falls off, the indicator light will light up to ensure that the ECG clip sensor is in good contact and the ECG signal collection can begin. The realization of pulse signal acquisition is to bundle the pulse sensor on the finger end and bundle the breath sensor with the waist of the human body to collect the breath signal. At the beginning of the signal collection, the subject should try to keep quiet and in a relaxed and natural posture to avoid signal changes caused by excessive fatigue or tension. Finally, the collected signal is transmitted to the host computer for storage. ECG, PPG, and RESP signals were collected from 25 subjects, including 14 females and 11 males. Ten groups of data were collected for 60 s for each subject, and a total of 250 data were collected to form the experimental dataset.

### 2.2. Prepare Knowledge and Algorithms

#### 2.2.1. Principal Component Analysis

Person et al. [21] proposed Principal Component Analysis (PCA) in 1901. It analyzes high-dimensional data, reduces dimensionality, and maximizes information by preserving the main data with the least dimension through linear transformation. Hypothesis data set: $X=(x_{1},x_{2},\ldots x_{n})$; the main steps of the PCA algorithm are as follows:

(1) Data are zeroed. Zero the mean of the data, subtract the sample mean $\bar{x}$ of all samples:
  (2) $$\bar{x}=\frac{1}{n}\sum_{n=1}^{n}x_{n}$$
(2) Calculate the covariance matrix J of the sample:
  (3) $$J=\frac{1}{n}\sum_{n=1}^{n}\left(x_{n}-\bar{x}\right){\left(x_{n}-\bar{x}\right)}^{T}=\frac{1}{n}XX^{T}$$
(3) Calculate the covariance matrix J eigenvalue and eigenvector.
(4) The eigenvector with the first k largest eigenvalues is normalized to form the eigenmatrix *D*.
(5) Calculate the new data Y after the dimensionality reduction of the sample, Y=DX.

#### 2.2.2. Linear Discriminant Analysis

Linear Discriminant Analysis (LDA) [22] is a commonly used dimensionality reduction method for supervised learning. LDA was developed based on PCA. Belhumeur. P.N. et al. [23] first proposed the LDA method. He compared the similarities and differences between PCA and LDA in detail. The biggest feature of LDA is that it can cover the feature information between each category so that the data after dimensionality reduction can be classified more easily. The basic idea is to map data into a low-dimensional space so that the distances between different classes are as large as possible and the distances between the same classes are as small as possible.

Hypothesis data set $D=\{(x_{1},y_{1}),(x_{2},y_{2}),\ldots ,(x_{m},y_{m})\}$, where x is a sample of any n-dimensional vector and y is the label of each sample, i=1,2…n. The main steps of the LDA algorithm are as follows:

(1) Calculate the divergence matrix $S_{w}$ within the sample class:
  (4) $$S_{w}=\sum_{0}+\sum_{1}=\sum_{x\epsilon x_{0}}{\left(x-u_{0})(x-u_{0}\right)}^{T}+\sum_{x\epsilon x_{1}}{\left(x-u_{1})(x-u_{1}\right)}^{T}$$

Including $\Sigma_{i}$ first class i sample covariance matrix, expression is $\Sigma_{i}=\sum_{x\epsilon x_{i}}{\left(x-u_{i})(x-u_{i}\right)}^{T}$, $u_{i}$ is the mean vector of the class i sample, $u_{i}=\frac{1}{n}\sum_{x\epsilon x_{i}}x$.

(2) Calculate the divergence matrix $S_{b}$ between sample classes:
  (5) $$S_{b}={\left(u_{0}-u_{1})(u_{0}-u_{1}\right)}^{T}$$

(3) Assuming the data set is reduced to k dimension, calculate
  (6) $$arg{max}_{w}J\left(W\right)=\frac{w^{T}S_{b}w}{w^{T}S_{w}w}$$

*J* (*w*) is to define the ratio of the distance between classes and the distance within classes. The maximum value of *J*(*w*) is the maximum eigenvalue of the matrix $S_{w}^{-1}S_{b}$, and the eigenvector of the maximum eigenvalue of $S_{w}^{-1}S_{b}$ is w. The K largest eigenvalues and the eigenvectors corresponding to the eigenvalues form the projection matrix W.

(4) The data on each sample characteristics $x_{i}$ into new samples $z_{i}=W^{T}x_{i}$, and $W^{\prime }=\{(z_{1},y_{1}),(z_{2},y_{2}),\ldots ,(z_{m},y_{m})\}$ are the latest data after dimensionality reduction.

#### 2.2.3. Canonical Correlation Analysis

Canonical Correlation analysis (CCA) [24] is a multivariate statistical analysis method that uses the correlation between comprehensive variable pairs to reflect the overall correlation between two groups of indicators. To grasp the correlation between the two groups of indicators as a whole, two representative comprehensive variables $U_{1}$ and $V_{1}$ (each is a linear combination of variables in two variable groups) are extracted from the two groups of variables, and the correlation between the two comprehensive variables is used to reflect the overall correlation between the two groups of indicators [25]. Therefore, based on the theory of CCA, Multiset Canonical Correlation Analysis (MCCA) [26] is proposed, which extends two groups of variables to multiple groups of variables to explore the correlation between multi-modal data. In more than one set of $X_{1},X_{2},\ldots X_{n}$ samples, it is assumed that each set of sample dimensions for $m_{i}(i=1,2\ldots n)$ and the samples in each set follow a Gaussian distribution and are centered. Define the criterion function of multiset canonical correlation analysis:

(7) $$J_{MCCA}\left(\alpha_{1},\alpha_{2},{\ldots \alpha }_{n}\right)=\frac{\sum_{i=1}^{n}\sum_{j=1}^{n}\alpha_{i}^{T}S_{ij}\alpha_{j}}{\sqrt{\sum_{i=1}^{n}\alpha_{i}^{T}S_{ii}\alpha_{i}}}\;$$

*α* is the correlation projection direction sought by each mode, which enables the projected multi-modal data to have the greatest inter-modal correlation. $S_{ij}=E\left[X_{i}X_{j}^{T}\right]$ is the $X_{i}$ and $X_{j}$ mutual covariance matrix. $S_{ii}$ is $X_{i}$ covariance matrix.

The above formula can be converted to the solution of the following problem:

(8) $$\underset{\alpha_{1},\alpha_{2}\ldots \alpha_{n}}{\max}\sum_{i=1}^{n}\sum_{j=1}^{n}\alpha_{i}^{T}S_{ij}\alpha_{j}\;s.t.\sum_{i=1}^{n}\alpha_{i}^{T}S_{ii}\alpha_{i}=1$$

The optimization problem of Formula (8) can be solved by constructing the Lagrange multiplier method:

(9) $$L\left(\alpha_{1},\alpha_{2},{\ldots \alpha }_{n}\right)=\sum_{i=1}^{n}\sum_{j=1}^{n}\alpha_{i}^{T}S_{ij}\alpha_{j}-\lambda (\sum_{i=1}^{n}\alpha_{i}^{T}S_{ii}\alpha_{i}-1)$$

*λ* is the Lagrange multiplier. Make$\;\partial L/\partial \alpha_{i}=0,i=1,2,\ldots n$, obtain $\sum_{j=1}^{n}S_{ij}\alpha_{j}=\lambda S_{ii}\alpha_{i}$, i=1,2,…n; Formula (9) can be equivalent to the following:

(10) $$\left[\begin{array}{ccc} \begin{array}{c} S_{11}\\ S_{21} \end{array} & \begin{array}{c} S_{12}\\ S_{22} \end{array} & \begin{array}{c} \begin{array}{cc} \cdots & S_{1n} \end{array}\\ \begin{array}{cc} \cdots & S_{2n} \end{array} \end{array}\\ \vdots & \vdots & \begin{array}{cc} \ddots & \vdots \end{array}\\ S_{n1} & S_{n2} & \begin{array}{cc} \ldots & S_{nn} \end{array} \end{array}\right]\left[\begin{array}{c} \begin{array}{c} \alpha_{1}\\ \alpha_{2} \end{array}\\ \begin{array}{c} \vdots \\ \alpha_{n} \end{array} \end{array}\right]=\lambda \left[\begin{array}{ccc} \begin{array}{c} S_{11}\\ 0 \end{array} & \begin{array}{c} 0\\ S_{22} \end{array} & \begin{array}{c} \begin{array}{cc} \cdots & 0 \end{array}\\ \begin{array}{cc} \cdots & 0 \end{array} \end{array}\\ \vdots & \vdots & \begin{array}{cc} \ddots & \vdots \end{array}\\ 0 & 0 & \begin{array}{cc} \ldots & S_{nn} \end{array} \end{array}\right]\left[\begin{array}{c} \begin{array}{c} \alpha_{1}\\ \alpha_{2} \end{array}\\ \begin{array}{c} \vdots \\ \alpha_{n} \end{array} \end{array}\right]$$

Formula (10) is a generalized eigenvalue problem. By solving it, multiple sets of eigenvectors corresponding to the largest eigenvalue K can be obtained, and M projection matrices $X_{i}^{\prime }$ co-responding to $X_{i}$ can be formed.

#### 2.2.4. Support Vector Machine (SVM)

SVM (Support Vector Machine) [27] is a binary classification supervised learning model that is applied to statistical classification and regression analysis. The basic model is defined as the linear classifier with the most significant spacing on the feature space, and the basic idea is to solve the separation hyperplane that can correctly partition the training data set and has the most critical geometric spacing. Suppose you are given a data set on a feature space $D=\{(x_{1},y_{1}),(x_{2},y_{2}),\ldots ,(x_{m},y_{m})\}$, $\;x_{i}\in R^{n}$,${\;y}_{m}\in \{+1,-1\}$, i=1,2,…m.

Construct the Lagrange function:

(11) $$\underset{\alpha }{\max}=\sum_{i=1}^{N}\alpha_{i}-\frac{1}{2}\sum_{i=1}^{N}\sum_{i=1}^{N}\alpha_{i}\alpha_{j}y_{i}y_{j}\left(x_{i}{\cdot y}_{i}\right)$$

Convert hyperplane to solve the dual-solve problem.

Hyperplane solution formula:

(12) $$min\delta \left(w\right)=\frac{1}{2}{\left\|w\right\|}^{2}+C\sum_{i=1}^{n}\xi_{i}\xi_{i}\geq 0,i=1,2,\ldots ,N$$

Including $\xi_{i}$ for slack variables and C for penalty parameters.

SVM commonly used kernel functions:

Linear kernel function:

(13) $$k(x_{i},x_{j})={x_{i}}^{T}x_{j}$$

Polynomial kernel function:

(14) $$k\left(x_{I},x_{j}\right)={\left({x_{i}}^{T}x_{j}\right)}^{d}$$

Gaussian kernel function:

(15) $$k\left(x_{I},x_{j}\right)=\exp\left(-\frac{\left\|x_{i}-x_{j}\right\|}{2\delta^{2}}\right)$$

Tangent hyperbolic kernel function:

(16) $$K\left(x,y\right)=\tanh\left[\beta \left(x*y\right)+k\right]$$

#### 2.2.5. Long Short-Term Memory (LSTM)

Long Short-Term Memory Neural Network (LSTM) [28] is a kind of recurrent neural network (RNN) that is used to solve the problem of long-term dependence commonly existing in general recurrent neural networks. LSTM can effectively convey and express information in a long time series and will not lead to the neglect (forgetting) of helpful information from a long time ago. It is widely used for time domain feature extraction of time series data. LSTMs are well suited to capture repetitive features from biomedical signals. Biomedical signals are typical time series data.

### 2.3. Data Preprocessing and Feature Extraction

**Figure 3:**
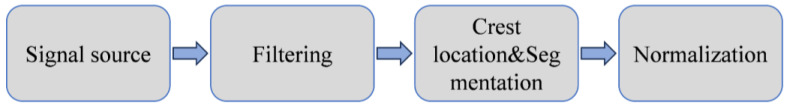
Block diagram of signal preprocessing.

The process of biomedical signal preprocessing and feature extraction is shown in Figure 3.

**Figure 4:**
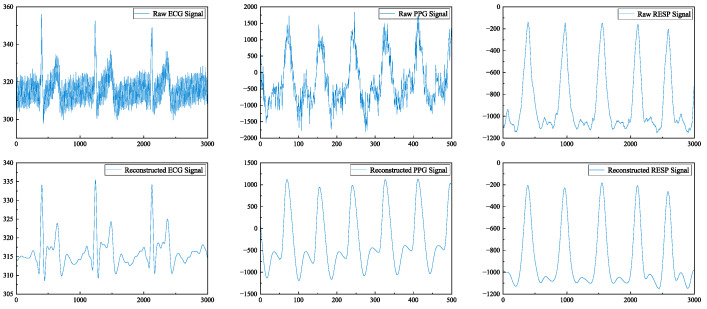
Comparison of biomedical signals before and after filtering.

**Figure 5:**
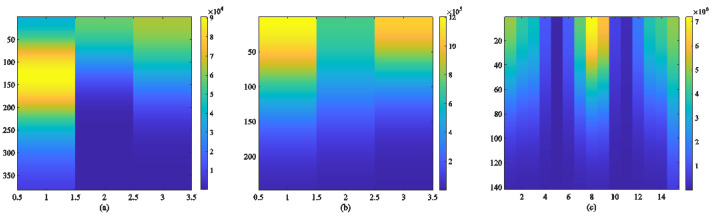
Energy spectrum diagram of (**a**) ECG, (**b**) PPG, and (**c**) RESP signals.

Biomedical signals are periodic nonlinear signals. During the measurement process, various interferences, such as myoelectric interference, power frequency interference, and baseline drift, can occur due to external environmental factors, the complexity of the tested individual, and equipment limitations. The preprocessing of data sets aims to clean the data by removing abnormal signals and noise interference and eliminating missing or invalid signals. Wavelet transform is a widely used method for denoising biomedical signals [29], which proves highly effective in denoising, compressing, and classifying non-stationary signals. By decomposing the signal on multiple scales [30] and extracting detail coefficients along with approximate coefficients, wavelet transform allows setting a threshold to remove detail coefficients containing noise while retaining useful information from biomedical signals through the reconstruction of wavelet coefficients [31]. In our previous work on wavelet denoising tests for biomedical signals using different wavelet functions, we determined that “db4” combined with the soft threshold denoising method was most suitable. The “db4” wavelet exhibits waveform similarities with ECG and PPG signals, thereby minimizing potential damage caused by wavelet reconstruction to both signal forms. The noise interference of the RESP signal in this study is minimal, and an FIR low-pass filter is utilized to effectively eliminate any interference present in the RESP signal, thereby obtaining a smooth and clear output signal. Figure 4 and Figure 5 illustrate the energy spectra of biomedical signals before pre-treatment as well as after preprocessing steps, including filtering.

**Figure 6:**
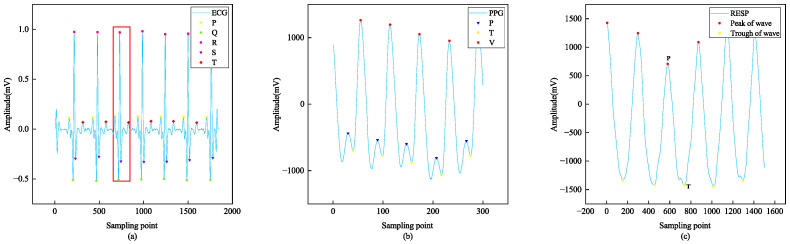
Localized waves of (**a**) ECG, (**b**) PPG, and (**c**) RESP signals. The waveform in the red box in (**a**) is a complete single-period beat.

The reference point feature extraction of biomedical signal waveform segmentation after pretreatment is illustrated in Figure 6. Reference point segmentation refers to the method used for dividing the heartbeat [32]. To perform waveform segmentation of ECG signals, it is necessary to detect the R-peak. To achieve this objective, various algorithms have been developed, and our method employs the well-established Pan–Tompkins algorithm [33].

In the QRS wave, the Q wave and S wave represent troughs occurring before and after the R wave, respectively. The Q and R waves can be determined by identifying two minimum points. By selecting sampling point T1 to include the duration of the P wave on the left side of the R wave peak and sampling point T2 to include the duration of the T wave on the right side, we can locate five reference points (P, Q, R, S, and T) in an ECG signal. This allows for the unification of QRS waveform data. The findpeaks function is capable of locating peak Y for each P wave with its corresponding position X in a PPG signal.

**Figure 7:**
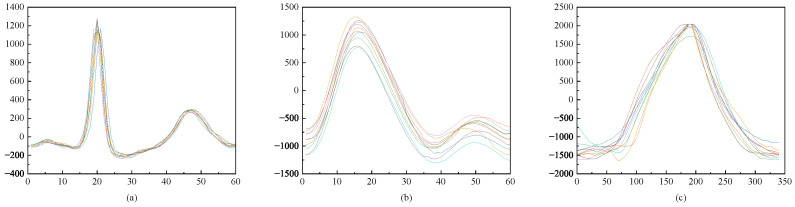
Complex vectors of (**a**) ECG, (**b**) PPG, and (**c**) RESP signals.

Additionally, it can calculate the minimum position between peaks and troughs to divide complete single-period PPG signals that encompass P waves, V waves, and starting and ending points. The RESP signal contains a single peak point, which can be identified where its derivative is zero or near zero if not precisely zero. Derivative methods are employed to locate both peaks and troughs within a RESP signal waveform segment. After segmenting a single periodic signal waveform, individual waveform complexes representing ECG, PPG, and RESP were generated, respectively. Figure 7 illustrates these results.

The composite waveform demonstrates the variation and correlation of different waveform features at an individual level, thereby ensuring the accurate capture and analysis of biomedical signals using the aforementioned method. The dimensionality of each signal sample is substantial, and the presence of redundant information does not contribute to identification but somewhat hampers the accuracy of the identification system. Time–frequency domain feature extraction is performed on the single-cycle signal waveforms of the three signals, with a total of 20 features extracted from each signal, including 15 time domain features and 5 frequency domain features. In this study, PCA and LDA techniques are employed to eliminate correlations among high-dimensional data and synthesize linearly independent low-dimensional data with minimal redundancy. This approach establishes a foundation for subsequent recognition that is both accurate and effective.

### 2.4. Dimensionality Reduction Fusion

**Figure 8:**
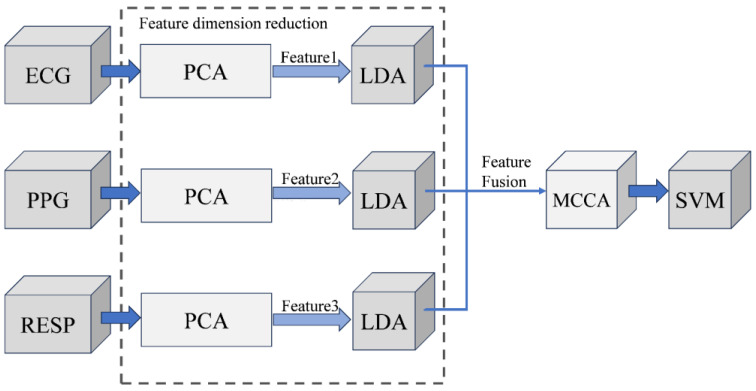
Block diagram of feature dimension reduction fusion.

The purpose of feature fusion is to establish a connection between the features extracted from the three biomedical signals through some mechanism and finally form a feature vector with multiple information, make up for the inherent defects of a single feature, achieve feature complementarity, have more identity recognition ability, and improve the recognition accuracy. The whole process is shown in Figure 8.

PCA and LDA were used to reduce the dimensionality of three signal feature matrices. Specifically, the eigenmatrices X, Y, and Z of ECG, PPG, and RESP signals were subjected to zeroization through PCA individually, resulting in obtaining eigenvectors from the standardized covariance matrix J. The contribution rate and cumulative contribution rate of each principal component were calculated, and only the first k principal components with high contribution rates containing adequate information were selected. These selected principal components were then synthesized into an eigenmatrix to obtain new dimensionality-reduced data while reducing the original eigenvector from n dimensions to k dimensions. Subsequently, LDA was applied for further dimension reduction by adjusting the discrete degree between classes for each type of signal separately. K is obtained by Formula (6) eigenvalues and corresponding eigenvectors of the projection matrix W, generating new sample $z_{i}=W^{T}x_{i}$, and finally, the characteristics of the data $W^{\prime },\;$respectively. Currently, three primary approaches exist to multi-modal data fusion, namely data layer fusion, feature layer fusion, and decision layer fusion. The MCCA fusion method is employed in this study for feature layer fusion, which involves combining two or more groups of appropriate features extracted from biometric data into a suitable feature vector. By incorporating depth information through this fusion process, the resulting feature vector accurately represents the biometric features and leverages the complementary advantages of multiple features. Commonly used algorithms for data feature layer fusion include parallel feature fusion, serial feature fusion, Gaussian mixture model [34], and Bayesian decision theory [35], among others. Serial feature layer fusion strategy: A new feature vector is formed by merging the beginning and end of the feature vector. Suppose you have two sets of feature vectors $x\in R^{n}$ and $y\in R^{n}$, then the characteristic vector of serial merge for z=xy and$\;z=R^{n+m}$. Parallel feature layer fusion strategy: combine two feature vectors into a parallel unit or compound vector. Suppose two sets of eigenvectors for the imaginary part and the fundamental part x and y; then, the compound eigenvector is expressed as *z*=x+iy, where i is the imaginary part unit. After performing dimensionality reduction on each biomedical signal to extract features, we employ the Lagrange multiplier method defined by MCCA (Formula (9)) to solve for the eigenvalues and corresponding eigenvectors. From these eigenvectors, we select the first k groups to construct a representative projection matrix D, which is then utilized to generate a new fusion feature set. Finally, identification is performed using an SVM classifier with optimized kernel parameters c and g.

## 3. Results

This section describes our experimental design and results. We have carried out three kinds of biomedical signal identification experiments: single-mode, double-mode, and multi-mode. Three types of experiments are trained and tested by using the fusion method of dimensionality reduction mentioned above, and the performance accuracy is evaluated by adjusting parameters and running at least 10 experiments.

The first kind of single-mode identification experiment verifies the feasibility of ECG, PPG, and RESP signal identification. Three signals of 25 individuals were selected from the MIMIC data set in Section 2.1. In the single-mode identification of ECG signals, the ECG signals of each individual were numbered from 1 to 25, and 20 groups of samples were extracted. Each sample contains 15 time domain features and 5 frequency domain features, forming a sample set with a size of 500. The same is true for PPG and RESP signals. The three sample sets are classified and identified by SVM. Before the identification, the sample set was randomly divided into the training set and the test set according to the ratio of 7:3. The algorithm marks the data of the identified user as the authentication user sample and regards all other users as intrusion saboteurs. Selecting polynomial kernel SVM for classification can also work better when the data are small, and the accuracy of the model to authenticate user characteristics is enhanced by multiple iterative training of SVM. The accuracy of single-modal identification is presented in Table 1.

**Figure 9:**
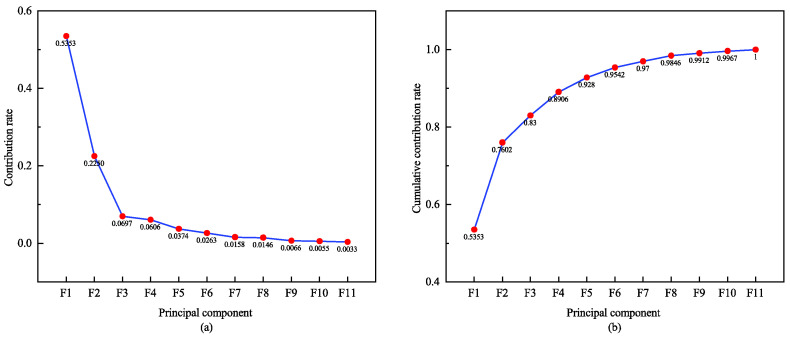
Principal component contribution rate and cumulative contribution rate of ECG. (**a**) shows the contribution rates of the first ten feature principal components for PCA reduction of ECG, and (**b**) shows the cumulative contribution rates of the first ten feature principal components.

**Figure 10:**
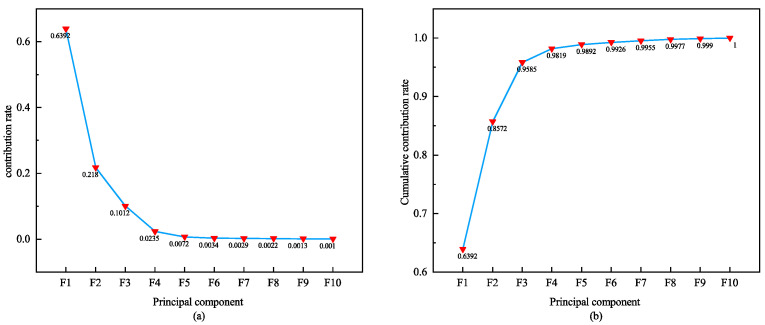
Principal component contribution rate and cumulative contribution rate of PPG. (**a**) shows the contribution rates of the first ten feature principal components for PCA reduction of PPG, and (**b**) shows the cumulative contribution rates of the first ten feature principal components.

**Figure 11:**
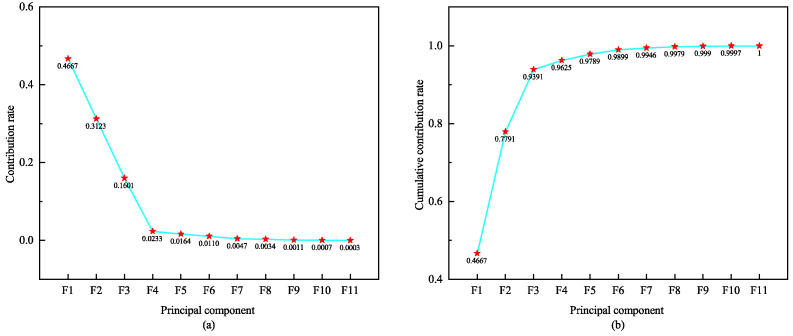
Principal component contribution rate and cumulative contribution rate of RESP. (**a**) shows the contribution rates of the first ten feature principal components for PCA reduction of RESP, and (**b**) shows the cumulative contribution rates of the first ten feature principal components.

To verify the scalability of signal fusion and the effectiveness of the proposed dimensionality reduction method, we conducted a second type of two-modal experiment. Firstly, the PCA algorithm is utilized to reduce the dimensionality of the three signals. By evaluating the contribution rate and cumulative contribution rate of each signal’s principal components, we can assess their performance in retaining information. A higher contribution rate indicates more excellent information retention by the principal component. Taking the ECG signal as an example, Figure 9 illustrates the contribution rate and cumulative contribution rate of its first 11 principal components. It is evident that while the contribution rate gradually decreases for each principal component, the cumulative contribution rate steadily increases. Upon reaching the tenth principal component, a cumulative contribution rate of 99.67% is achieved. This implies that with only 10 dimensions, the post-PCA reduction ECG feature vector can already represent a pre-dimensionality reduced feature vector with a high 99.67% contribution rate; further increasing dimensions brings minimal additional contributions. Therefore, the effective dimensionality reduction of ECG features is successfully achieved by using the 10-D ECG vector after PCA fusion. Similarly, PCA dimensionality reduction is performed on eigenvectors obtained from PPG and RESP signals, respectively. Figure 9, Figure 10 and Figure 11 display the respective principal component’s contribution rates and cumulative contributions rates after PCA dimensionality reduction for these three signals. These results confirm efficacy in multiple signals of PCA-based dimensional reductions, thereby ensuring efficient and accurate data processing.

Different feature fusion methods and training algorithms were used to perform dual-mode fusion of ECG and PPG, and the results are shown in Table 2. The specific fusion methods include parallel, serial, and CCA fusion.

The first method uses parallel, serial, and CCA fusion for ECG and PPG signal sample sets with feature extraction but no dimensionality reduction, respectively, and identifies and verifies them on LSTM and SVM.

In the second method, we use CCA to fuse the ECG and PPG signal feature vectors after PCA dimension reduction.

The two methods were identified on the MIMIC data set, and the self-collected data set of the Pclab-801 embedded biomedical electronic laboratory in Section 2.1. Each individual contains 20 sets of samples, numbered from 1 to 25 by individual, to form two sets of 500 samples each—the public set and the self-sampled set. The training set and the test set were randomly divided according to 7:3, and the individual was identified. CCA is a statistical analysis method used to study the correlation between two groups of variables, and it is also an effective dimensionality reduction technique that performs well in fusing two groups of variables. The experimental results show that compared with serial, parallel, and CCA fusion experiments without dimensionality reduction, the scheme combined with PCA reduction and CCA fusion is more significant in improving the recognition accuracy. This method shows stable and excellent performance on both public and self-collected data sets and can effectively improve the accuracy of multi-modal identity recognition. At the same time, the experimental data also show that in our serial and parallel fusion recognition experiments on unidimensionally reduced signals, the accuracy of LSTM is relatively low in the face of the same data set, which may be due to the high complexity of LSTM training and optimization. In addition, LSTM has a greater demand on data volume and computational resources, and our sample set may result in the LSTM model not generalizing well to the validation set or the test set. SVM as a classifier has a slight advantage in recognition accuracy. Because of its inherent regularization characteristics, SVM is not easy to overfit and can maintain a high accuracy on the training set and test set of small samples. Especially in the processing of complex multi-modal physiological signals, the SVM classification effect is more obvious. The results provide a solid theoretical and experimental basis for further optimization of the classification model and selection of SVM for three-mode identity recognition in our subsequent work.

**Figure 12:**
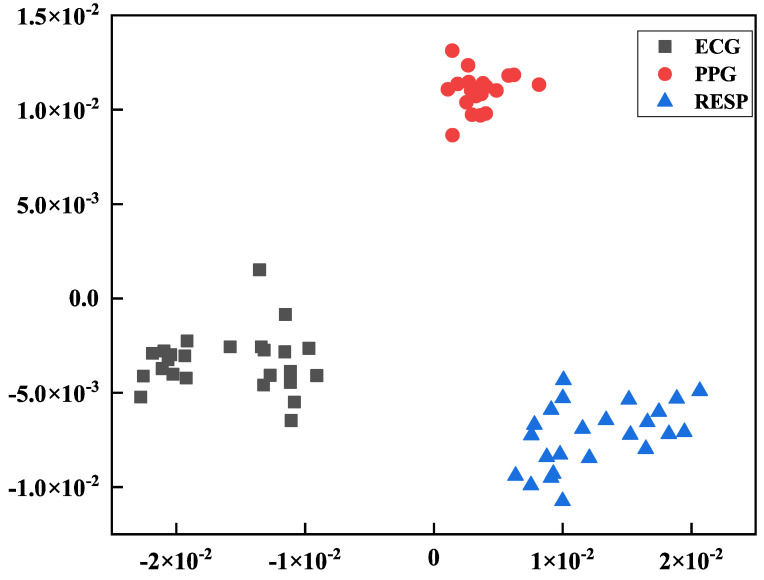
LDA performs secondary dimensionality reduction results.

We add RESP signals into the dimensionality reduction fusion and use three kinds of signals to carry out a more in-depth third kind of multi-mode fusion experiment. Similarly, experiments were carried out on the MIMIC data set and a self-collected data set of the Pclab-801 embedded biomedical electronic experiment box, respectively, and three signals of 25 individual data were fused and identified. Consistent with the above feature extraction, dimensionality reduction processing, and sample set composition methods, on the basis of the initial dimensionality reduction of the three physiological signals by PCA, we further reduced the dimensionality of the three signals to six dimensions by using LDA to create a smaller dimensionality reduction fusion sample dataset. LDA performs lower dimension reduction and can also distinguish the categories of the three signals well, as shown in Figure 12.

Finally, the feature matrix of the three signals after dimensionality reduction is fused using MCCA. Through this multi-step dimensionality reduction and multi-modal fusion method, we can effectively reduce the dimensionality of the data while preserving the critical classification information. The identity verification is also carried out on the MIMI data set and the self-collected data set. Both datasets contain 25 individuals, each containing 20 groups of fused samples. The training and testing of 500 samples are randomly divided by 7:3. We use SVM to repeat classification recognition verification 10 times on a random test set and average the individual recognition accuracy of 10 tests to determine the total performance of identity recognition. The results are shown in Table 3. Using the method described in Section 4, the accuracy rate is effectively improved, and the overall recognition rate of the public data set and self-collected data set reaches more than 99% accuracy, which was 99.56% and 99.69%, respectively. Compared with the previous two experimental results, the recognition effect of three-mode dimensionality reduction fusion features is better than that of single-mode and dual-mode.

**Figure 13:**
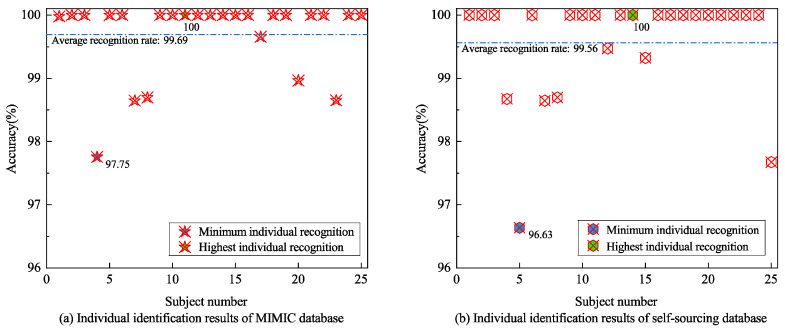
Individual identification results.

We show the individual recognition authentication results of MIMIC data sets and self-collected data sets, as shown in Figure 13. The lowest individual recognition accuracy was 97.75% in the mimic data set and 96.63% in the self-collected data set. More than half of the recognition rates reached 100%. From the perspective of physiological signal data, the lower accuracy rate of individual physiological signal identification than the average recognition rate may be caused by the following factors: First, individual differences. There are natural differences in the physiological characteristics of different individuals, which increases the difficulty of feature extraction and makes it difficult for the model to capture their unique features, which affects the accuracy effectively. Second, signal volatility and instability. Emotion, fatigue state, environment, and other factors will lead to large fluctuations in the signal collection process, increasing the difficulty of identification. Third, noise interference. Physiological signals (such as breathing, etc.) are susceptible to external interference, artifacts caused by the movement of the subject, or physiological noise, which will reduce the quality of the signal, thus affecting recognition accuracy. The process of physiological signal acquisition and recognition itself has a specific correlation, and these factors are inevitable. Through this individual-level identification test, we can further ensure the reliability of the method in practical applications.

In Table 4, we present a comparative analysis of the existing literature. The results indicate that our identity integrating features from various biomedical signals has effectively improved both the precision and stability of identification, which has been thoroughly validated by the experimental outcomes.

## 4. Conclusions

This study introduces an identity recognition method based on the dimensionality reduction and fusion of multi-modal biomedical signal features. In the research, we have carried out three types of biomedical signal identification: single-mode, double-mode, and multi-mode. Results demonstrate that the combination of PCA and LDA effectively reduces the dimension of these signals’ data. Moreover, employing MCCA for feature fusion yields superior accuracy and recognition outcomes compared to other methods used in multi-modal biomedical signal identification. Furthermore, the study reveals that identity recognition from biomedical signals remains a challenging task. While incorporating three input sources for feature fusion enhances identity recognition accuracy in this study, future research should consider collecting biomedical signals under different states (e.g., motion state and tension state) to achieve more robust and reliable results. ECG, PPG, and RESP were chosen as preferred biomedical signals due to their ease of collection using ergonomic wearable devices. The purpose of both single-modal and multi-modal identification experiments is to validate the suitability of biomedical signals for recognition. In the future, this technology could be applied in remote monitoring systems or intelligent wearable devices to identify and monitor the health status of essential populations, such as elderly individuals receiving home care or residing in nursing homes, thereby ensuring prompt healthcare intervention in case of emergencies.

## Figures and Tables

**Table 1:** Single-mode identification results.

Database	ECG	PPG	RESP
MIMIC	87.5%	79.87%	74.84%

**Table 2:** ECG and PPG bimodal biomedical signals recognition results.

Method	MIMIC	Self-Sourcing
Series fusion + SVM	94.87%	96.80%
Series fusion + LSTM	90.62%	93.75%
Parallel fusion + SVM	92.27%	92.50%
Parallel fusion + LSTM	92.74%	93.64%
CCA + SVM	97.43%	97.62%
PCA + CCA + SVM	96.20%	97.77%

**Table 3:** Results of three-mode biomedical signal identification.

Method	MIMIC	Self-Sourcing
PCA + LDA + MCCA + SVM	99.56%	99.69%

**Table 4:** Compared with the existing research literature.

Study	Method	Feature	Results
[15]	Cross-Correlation Function (CCF)	ECG, PPG	Accuracy: PPG, 99.98%; ECG, 88.79%
[16]	Score Fusion	ECG, Fingerprint	Accuracy: ECG, 90.0%; ECG + Fingerprint, EER:0.1%
[36]	CNN	ECG, PPG	Accuracy: 98.9%
[13]	CNN, WPT, AR, IF, and SE	ECG, PPG	Accuracy: 97.5%
This work	PCA, LDA, MCCA, SVM	ECG, PPG, RESP	Accuracy: MIMIC, 99.56% Self-sourcing: 99.69%

## Data Availability

The data presented in this study are available on request from the corresponding author.

## Abbreviations

The following abbreviations are used in this manuscript:

ECG	Electrocardiogram
PPG	Photoelectric Plethysmography
RESP	Respiratory
PCA	Principal Component Analysis
LDA	Linear Discriminant Analysis
CCA	Canonical Correlation Analysis
MCCA	Multiset Canonical Correlation Analysis
A/D	Analog to Digital
LCD	Liquid Crystal Display
db4	Dobessie wavelet of order 4
SVM	Support Vector Machine
LSTM	Long Short-Term Memory

## References

[1] Kinsella K.G., Kinsella D.R. (2005). Global aging: The challenge of success. Popul. Bull..

[2] UN. Department of Economic and Social Affairs (2023). World Social Report 2023: Leaving No One Behind in an Ageing World.

[3] Zhang Y., Gu Z., Xu Y., He M., Gerber B.S., Wang Z., Liu F., Peng C. (2023). Global scientific trends in healthy aging in the early 21st century: A data-driven scientometric and visualized analysis. Heliyon.

[4] Sancho J., Iglesias Á., García J. (2018). Biometric Authentication Using the PPG: A Long-Term Feasibility Study. Sensors.

[5] Li H., Wang X.P., Liu C., Li P., Jiao Y. (2021). Integrating multi-domain deep features of electrocardiogram and phonocardiogram for coronary artery disease detection. Comput. Biol. Med..

[6] Everson L., Biswas D., Panwar M., Rodopoulos D., Helleputte N.V. BiometricNet: Deep Learning based Biometric Identification using Wrist-Worn PPG. Proceedings of the 2018 IEEE International Symposium on Circuits and Systems (ISCAS).

[7] Li Y.Z., Pang Y.W., Wang K.Q., Li X.L. (2020). Toward improving ECG biometric identification using cascaded convolutional neural networks. Neurocomputing.

[8] Hammad M., Liu Y., Wang K. (2019). Multimodal Biometric Authentication Systems Using Convolution Neural Network Based on Different Level Fusion of ECG and Fingerprint. IEEE Access.

[9] Ahamed F., Farid F., Suleiman B., Jan Z., Wahsheh L.A., Shahrestani S. (2022). An Intelligent Multimodal Biometric Authentication Model for Personalised Healthcare Services. Future Internet.

[10] Bastos L., Tavares T., Tavares D.D., Cerqueira E., Santos A.L., Lima M.N. Double Authentication Model based on PPG and ECG Signals. Proceedings of the 2020 International Wireless Communications and Mobile Computing (IWCMC).

[11] Yaacoubi C., Besrour R., Lachiri Z. A multimodal biometric identification system based on ECG and PPG signals. Proceedings of the 2nd International Conference on Digital Tools & Uses Congress.

[12] El-Rahiem B.A., El-Samie F., Amin M. (2021). Multimodal biometric authentication based on deep fusion of electrocardiogram (ECG) and finger vein. Multimed. Syst..

[13] Alkeem E.A., Yeun C.Y., Yun J., Yoo P., Chae M., Rahman A. (2021). Robust Deep Identification using ECG and Multimodal Biometrics for Industrial Internet of Things. Ad. Hoc. Netw..

[14] GirishRaoSalankeN S., Maheswari N.U., Samraj A., Vijayakumar M. (2018). An Efficient Score level Multimodal Biometric System using ECG and Fingerprint. J. Telecommun. Electron. Comput. Eng..

[15] Tatar A.B. (2023). Biometric identification system using EEG signals. Neural Comput. Appl..

[16] Aleidan A.A., Abbas Q., Daadaa Y., Qureshi I., Perumal G., Ibrahim M.E., Ahmed A.E. (2023). Biometric-Based Human Identification Using Ensemble-Based Technique and ECG Signals. Appl. Sci..

[17] Coelho K.K., Tristão E.T., Nogueira M., Vieira A.B., Nacif J.A.M. (2023). Multimodal biometric authentication method by federated learning. Biomed. Signal Process. Control.

[18] El_Rahman S.A., Alluhaidan A.S. (2024). Enhanced multimodal biometric recognition systems based on deep learning and traditional methods in smart environments. PLoS ONE.

[19] Moody G.B., Mark R.G. A database to support development and evaluation of intelligent intensive care monitoring. Proceedings of the Computers in Cardiology 1996.

[20] Goldberger A.L., Amaral L.A., Glass L., Hausdorff J.M., Ivanov P.C., Mark R.G., Mietus J.E., Moody G.B., Peng C.-K., Stanley H.E. (2000). PhysioBank, PhysioToolkit, and PhysioNet: Components of a new research resource for complex physiologic signals. Circulation.

[21] Pearson K. (1901). LIII. On lines and planes of closest fit to systems of points in space. Philos. Mag. Ser..

[22] Fisher R.A. (1936). The use of multiple measurements in taxonomic problems. Ann. Eugen..

[23] Belhumeur P.N., Hespanha J.P., Kriegman D.J. Eigenfaces vs. Fisherfaces: Recognition Using Class Specific Linear Projection. Proceedings of the Fourth European Conference on Computer Vision.

[24] Mohammadi-Nejad A., Hossein-Zadeh G., Soltanian-Zadeh H. (2017). Structured and Sparse Canonical Correlation Analysis as a Brain-Wide Multi-Modal Data Fusion Approach. IEEE Trans. Med. Imaging.

[25] Kettenring J.R. (1971). Canonical Analysis of Several Sets of Variables. Biometrika.

[26] Vapnik V.N. (1999). An overview of statistical learning theory. IEEE Trans. Neural Netw..

[27] Mukherjee S., Rifkin R.M. Support Vector Machine Classification of Microarray Data. *Computer Science*, Biology, 2001. https://www.researchgate.net/publication/2597477_Support_Vector_Machine_Classification_of_Microarray_Data.

[28] Hochreiter S., Schmidhuber J. (1997). Long Short-Term Memory. Neural Comput..

[29] Rioul O., Vetterlim M. (1991). Wavelets and signal processing. IEEE Signal Process. Mag..

[30] Mallat S.G. (1989). A Theory for Multiresolution Signal Decomposition: The Wavelet Representation. IEEE Trans. Pattern Anal. Mach. Intell..

[31] Zhao Q.B., Zhao L.Q. ECG Feature Extraction and Classification Using Wavelet Transform and Support Vector Machines. Proceedings of the 2005 International Conference on Neural Networks and Brain.

[32] Chang H., Yeung D.T. (2006). Robust locally linear embedding. Pattern Recognit..

[33] Pan J., Tompkins W.J. (1985). A Real-Time QRS Detection Algorithm. IEEE Trans. Biomed. Eng..

[34] Yan J., Wang S., Xie T., Yang Y., Wang J.Y. (2017). Variational Bayesian learning for background subtraction based on local fusion feature. Let Comput. Vis..

[35] Thakur G.S. (2014). Deterministic Bayesian Information Fusion and the Analysis of its Performance. Inf. Inference A J. IMA.

[36] Mousavi F.S. (2020). Fusion of ECG and PPG Signals in Apply to Spoof Detection and Biometric Authentication. Master’s Thesis.

